# Automated Decision-Support System for Prediction of Treatment Responders in Acute Ischemic Stroke

**DOI:** 10.3389/fneur.2013.00140

**Published:** 2013-09-26

**Authors:** Kartheeban Nagenthiraja, Brian P. Walcott, Mikkel B. Hansen, Leif Østergaard, Kim Mouridsen

**Affiliations:** ^1^Center of Functionally Integrative Neuroscience and MINDLab, Aarhus University, Aarhus, Denmark; ^2^Athinoula A. Martinos Center for Biomedical Imaging, Massachusetts General Hospital and Harvard Medical School, Boston, MA, USA; ^3^Department of Neurosurgery, Massachusetts General Hospital and Harvard Medical School, Boston, MA, USA; ^4^Cardiovascular Research Center, Massachusetts General Hospital and Harvard Medical School, Charlestown, MA, USA

**Keywords:** stroke, brain edema, magnetic resonance imaging, brain ischemia, decision-support systems, clinical, thrombolytic therapy

## Abstract

MRI is widely used in the assessment of acute ischemic stroke. In particular, it identifies the mismatch between hypoperfused and the permanently damaged tissue, the PWI-DWI mismatch volume. It is used to help triage patients into active or supportive treatment pathways. COMBAT Stroke is an automated software tool for estimating the mismatch volume and ratio based on MRI. Herein, we validate the decision made by the software with actual clinical decision rendered. Furthermore, we evaluate the association between treatment decisions (both automated and actual) and outcomes. COMBAT Stroke was used to determine PWI-DWI mismatch volume and ratio in 228 patients from two European multi-center stroke databases. We performed confusion matrix analysis to summarize the agreement between the automated selection and the clinical decision. Finally, we evaluated the clinical and imaging outcomes of the patients in the four entries of the confusion matrix (true positive, true negative, false negative, and false positive). About 186 of 228 patients with acute stroke underwent thrombolytic treatment, with the remaining 42 receiving supportive treatment only. Selection based on radiographic criteria using COMBAT Stroke classified 142 patients as potential candidates for thrombolytic treatment and 86 for supportive treatment; 60% sensitivity and 29% specificity. The patients deemed eligible for thrombolytic treatment by COMBAT Stroke demonstrated significantly higher rates of compromised tissue salvage, less neurological deficit, and were more likely to experience thrombus dissolving and reestablishment of normal blood flow at 24 h follow-up compared to those who were treated without substantial PWI-DWI mismatch. These results provide evidence that COMBAT Stroke, in addition to clinical assessment, may offer an optimal framework for a fast, efficient, and standardized clinical support tool to select patients for thrombolysis in acute ischemic stroke.

## Introduction

Neuroimaging is valuable to identify acute ischemic stroke patients that may benefit from thrombolysis (1, 2). Magnetic resonance based diffusion- and perfusion-weighted imaging (DWI and PWI) are widely utilized modalities in clinical practice that aid in treatment selection (3). While DWI hyper-intense regions typically indicate cytotoxic edema as a surrogate for permanent tissue injury, delayed PWI regions correspond to tissue with compromised hemodynamics (4, 5).

The ischemic penumbra is defined as tissue that is hypoperfused to such an extent that focal neurological symptoms arise, but where neurological function can be restored and tissue survival is ensured by early reperfusion.

Current PWI techniques are not able to distinguish between critically hypoperfused tissue and benign oligemia. This shortcoming potentially overestimates the actual mismatch volume and is especially noticeable with small lesions (6). Additionally, hyper-intense areas on diffusion-weighted images have shown reversal following thrombolysis (7, 8). Nevertheless, the PWI-DWI mismatch is recognized as surrogate for risk at tissue that may be viable if perfusion is restored timely (4, 9, 10). Accordingly, the volume of the mismatch and the ratio between the volume of mismatch and volume of permanent injured tissue (PWI-DWI mismatch ratio) are two practical markers that are used to help triage stroke patients into interventional or supportive treatment pathways. For example, when considered along with other parameters, it has been shown that patients with a mismatch volume >10 mL and a PWI-DWI mismatch ratio >1.2 have greater benefit from thrombolysis compared to those without a significant mismatch (11–13). The time and expertise required to perform these calculations, along with inter-observer variability are potential factors that may limit their utilization and efficacy. To overcome these factors, automated computer-based algorithms for the determination of PWI-DWI mismatch have been developed (14). The overall aim of this study was to compare the agreement and outcomes between a solely automated computer-based patient triage algorithm and the actual clinical decision made for thrombolysis. Herein, we tested whether the treatment decisions of an automated patient selection software tool “Computer-Based Decision-Support System for Thrombolysis in Stroke” (COMBAT Stroke), were associated with 24 h clinical and imaging outcomes.

## Materials and Methods

### Patients and data acquisition

Following approval from national and regional ethics committees, patients with acute ischemic stroke were identified from the European I-Know consortium (2006–2009) and the Remote Ischemic Perconditioning Study (RIPS, 2009-2011) (15, 16). Clinical information available in the databases includes gender, age, time from symptom onset to treatment initiation, immediate and 24 h and 3 month follow-up National Institutes of Health Stroke Scale (NIHSS) score, lesion laterality, stroke etiology subtype, treatment (i.e., intravenous recombinant tissue plasminogen activator (rt-PA), or supportive treatment), admission blood pressure, presence of intracranial hemorrhage, home medications, platelet count, and MR angiography-based recanalization status at 24 h. Only adult patients (age >18) with acute ischemic stroke in the anterior circulation territory were included. Patients with ischemic strokes of the vertebrobasilar circulation were excluded.

Standard dynamic susceptibility contrast MRI was performed on various scanner types at different field strengths (GE Signa Excite 1.5T, GE Signa Excite 3T, GE Signa HDx 1.5T, GE Signa Horizon 1.5T, Siemens TrioTim 3T, Siemens Avanto 1.5T, Siemens Sonata 1.5T, Philips Gyroscan NT 1.5T, Phillips Achieva 1.5T, and Philips Intera 1.5T). The PWI sequence (TE 30/50 ms for 3 and 1.5 Tesla field strength, TR 1500 ms, FOV 24 cm, matrix 128 × 128, slice thickness 5 mm) was obtained during intravenous injection of Gadolinium based contrast (0.1 mmol/kg) at a rate of 5 mL/s followed by 30 mL of physiological saline at the same injection rate. Echo-planar DWI was obtained at *b*-value = 0 s/mm^2^ and *b*-value = 1.000 s/mm^2^. DWI images were automatically linear co-registered and re-sliced to acute PWI space using SPM8 (Wellcome Trust Centre for Neuroimaging, UCL, UK).

### Clinical practice

Patients form highly specialized stroke centers from Denmark, Germany, United Kingdom, France, and Spain were included in this study. The local rt-PA practices follow the general recommendation from American Heart Association, where patients older than 18 years with no imaging or clinical evidence for hemorrhage who have significant neurological deficit are eligible for treatment within 4.5 h of symptom onset. All centers routinely perform DWI and PWI in clinical practice and use the mismatch information as well as clinical information in treatment decision-making.

### Automatic mismatch estimation

COMBAT Stroke generates and applies a whole-brain mask to acute images to exclude non-brain structures, e.g., eyes, cerebrospinal fluid etc. Next, the software determines the lesion laterality by analyzing the acute apparent diffusion coefficient map (ADC) and the time-to-peak of the tissue curve map (TTP). The lesion laterality is used to compute a normal tissue reference and subsequently the algorithm outlines the initially injured tissue on DWI. Similarly, the algorithm automatically outlines the hypoperfused lesion on TTP map, and the mismatch is defined as the part of the hypoperfused lesion that is not contained in the co-registered DWI lesion. We automatically estimated the PWI-DWI mismatch ratio and mismatch volume for all patients in the cohort using COMBAT Stroke.

### Manual mismatch outlining

Comparing automated delineations of the mismatch to those determined manually by a single expert has the potential to introduce bias, therefore we used four expert raters (neuroradiologists) with extensive clinical experience in stroke diagnosis. The raters manually outlined the hypoperfused lesion on TTP maps and the lesion core on DWI images using in-house developed software. All raters worked individually and were blinded to each other and the remainder of the clinical data. Next, an expert rater consensus mismatch mask was created by summating the ratings (1 = lesion; 0 = normal) in every image voxel for each of the four expert raters. We utilized a cutoff summative score of 3 to create the mask to establish a consensus, consistent with previously described methods (14). The stroke community has been discussing the optimal choice of PWI map in stroke management, however no consensus exists. We chose TTP as the PWI modality because of its perceived superiority in separating normo- and hypoperfused tissue. A recent study demonstrated that TTP performed superior to deconvolved maps (e.g., MTT and Tmax) in terms of predicting tissue fate (17).

### Confusion matrix modeling

Initially, all patients were assessed exclusively on imaging criteria alone: thrombolysis could potentially be administered in cases were PWI-DWI mismatch ratio >1.2 and mismatch volume >10 mL. Assessments were made for each patient twice, once with the use of COMBAT Stroke and again by using the data generated by the expert neuroradiologists’ consensus. Next, confusion matrices were generated to quantify the performance of (1) COMBAT Stroke versus actual clinical decision and (2) COMBAT Stroke versus human measurement. The four entries of the confusion matrix denote true positive (TP, both clinical decision and COMBAT Stroke agree for thrombolytic therapy), true negative (TN, both clinical decision and COMBAT Stroke agree for supportive treatment), false positive (FP, COMBAT Stroke supports thrombolysis, but the patient was treated supportively), and false negative (FN, COMBAT Stroke advises for supportive treatment, however the patient was treated with thrombolysis).

**Figure 1:**
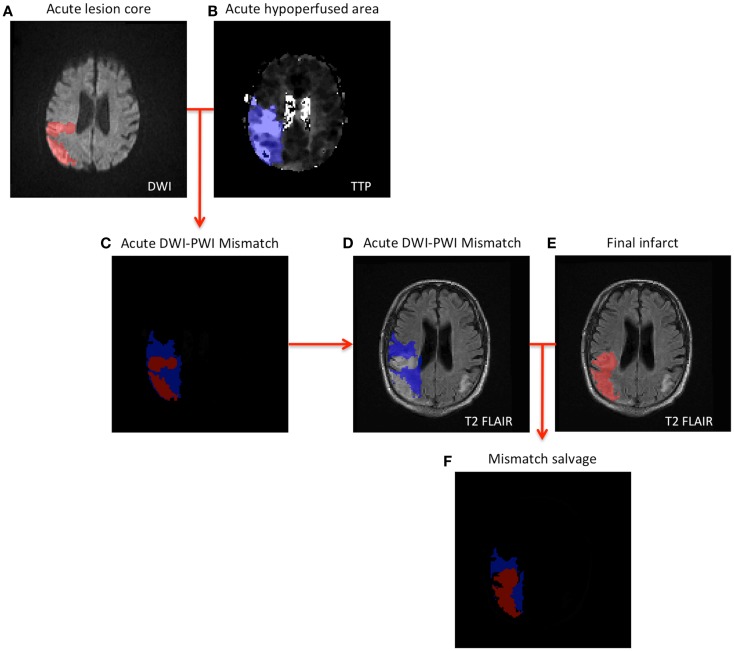
**Schematic illustration of mismatch salvage computation (A,B)**. The acute lesions are outlined on DWI and TTP **(C)**. Illustrates the concept of DWI-PWI mismatch (blue region) **(D–F)**. Combining the DWI-PWI mismatch information with the 1-month infarct evolution on T2 FLAIR yields the mismatch salvage (blue region in **F**).

Mindful that a multitude of factors are used in the actual clinical determination of administering thrombolytic treatment, we analyzed the following presentation variables for the four entries of the confusion matrix: mismatch volume, NIHSS score, and contraindications. The definition of contraindication in this study was based on the American Heart Association’s Stroke Guidelines and includes any of the following criteria: systolic blood pressure greater than 185 mmHg, diastolic blood pressure greater than 110 mmHg, evidence of intracranial hemorrhage, use of anticoagulants, international normalized ratio (INR) greater than 1.7, and time for symptom onset to treatment greater than 4.5 h, all of which were available in the database. Subsequently, we analyzed the clinical outcomes, in terms of mismatch salvage, reduction in NIHSS score at 24 h, and vessel recanalization determined by MR angiography at 24 h, to determine the association between presence/absence of significant acute mismatch and clinical outcome. The acute DWI-PWI mismatch was determined as the fraction of the hypoperfused area, which was not contained in the lesion core (Figure 1). Infarct evolution at 1 month follow-up was evaluated on T2 FLAIR for all patients in the cohort. The mismatch salvage was the portion of the acute mismatch that remained functional at 1 month follow-up. Rank sum-test was conducted to test the difference in median value of acute decision parameters and clinical outcomes between the groups.

To examine the relative importance of the presentation variables (Mismatch volume, NIHSS, and contraindications) in predicting thrombolytic treatment, we also conducted a classification and regression tree (CART) analysis. The best spilled of data at each node was determined by optimization of the Gini’s diversity index and the analysis was conducted in MATLAB 2010B (The MathWorks, Inc. Natick, MA, USA).

## Results

228 patients (136 males, 92 females) were identified as meeting criteria for analysis from the European I-Know consortium database and the Remote Ischemic Perconditioning Study. The patient characteristics for the two study populations are shown in Table 1. In comparison with the I-Know cohort the patients in the RIPS study had significantly lower neurological deficit at baseline, 24 h, and 3 month follow-up in terms of NIHSS and modified Rankin Score. No significant difference in the acute mismatch volume between the I-Know patients [56 mL, 25th and 75th percentile = (5, 133)] and RIPS patients [36 mL (1, 105)] was observed. 89% of the patients in the I-Know study were treated actively compared to 100% in the RIPS study. Altogether, 40 patients exceeded the 4.5-h treatment window, of whom 16 were treated actively. The median processing time for automatic PWI-DWI mismatch estimation across all patients was 31 s when processed on an Apple MacBook Pro, 2.6 GHz Intel Core i7.

**Table 1:** **Demographic and clinical characteristics of the patient cohort**.

	I-Know	RIPS	*p*
Patients	129	99	
Male	77 (60%)	59 (60%)	0.108
Median age and IQ range, years	70 (60, 77)	69 (61, 76)	0.922
Left hemisphere stroke	74 (57%)	50 (50%)	<0.001
IV t-PA	89 (69%)	99 (100%)	<0.001
Median time to treatment and IQ range, min	188 (150, 280)	164 (137, 220)	0.006
Time to treatment > 4.5 H	34 (26%)	6 (6%)	<0.001
Treated beyond 4.5 H	10 (29%)	6 (100%)	0.002
Median acute NIHSS and IQ range	11 (6, 16)	6 (4, 10)	<0.001
Median 24 h NIHSS and IQ range	4 (1, 10.25)	2 (1, 6)	<0.001
Median 3 months NIHSS and IQ range	2 (0, 6)	0 (0, 2)	<0.001
Median 3 months mRS and IQ range	2 (0, 3)	1 (0, 3)	<0.001
Median systolic BP and IQ range (mmHg)	145 (130, 158)	150 (140, 165)	0.038
Median diastolic blood pressure	83 (72, 90)	85 (79, 90)	0.406
Anticoagulant therapy	22 (17%)	1 (1%)	<0.001
Clopidogrel therapy	18 (14%)	6 (6%)	0.027
Median mismatch volume (mL)	56 (5, 133)	36 (1, 105)	0.179
Mismatch volume <10 mL	37 (29%)	33 (33%)	0.086
Mismatch volume <10 and >100 mL	51 (39%)	38 (38%)	0.031
Mismatch volume >100 mL	41 (32%)	28 (28%)	0.098
**STROKE SUB-TYPES**			
Cardiac source of emboli	58	36	0.046
Large vessel disease with significant carotid stenosis	22	16	0.140
Large vessel disease, other	12	10	0.174
Small vessel disease (lacunar)	5	24	<0.001
Carotid dissection	6	2	0.172
Other/unusual cause	3	1	0.315
Undetermined	23	10	0.039

Values represented as median with interquartile ranges presented in brackets. Sample size, where appropriate, is represented as numbers and the percentage of the cohort is contained in parentheses.

### Confusion matrix – combat versus clinical decision

**Figure 2:**
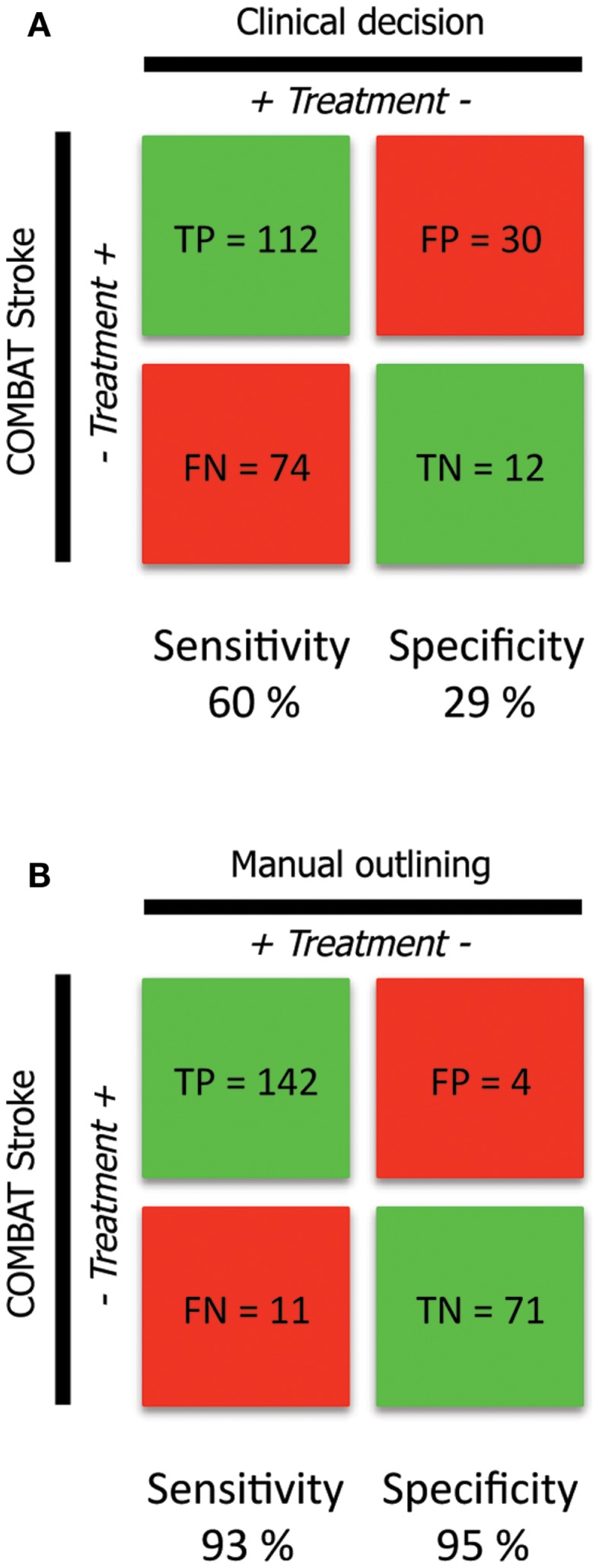
**Confusion matrix analysis (A)**. The confusion matrix demonstrates the agreement and disagreement between the clinical decisions actually rendered (columns) and automated patient selection based on radiographic criteria alone with the COMBAT Stroke software (rows) **(B)**. The confusion matrix demonstrates the agreement and disagreement between manual (columns) and automated (rows) calculations operating under the principle that thrombolysis should potentially be administered in cases were PWI-DWI mismatch ratio >1.2 and mismatch volume >10 mL.

186 of 228 patients underwent thrombolytic treatment, with the remaining 42 patients receiving supportive treatment only (Figure 2A, columns). Assessment of the cohort based on mismatch criteria (mismatch > 10 mL and PWI-DWI ratio > 1.2) alone with COMBAT Stroke classified 142 patients as potential candidates for thrombolytic treatment and 86 for supportive treatment; 60% sensitivity and 29% specificity, 79% positive predictive value, and 14% negative predictive value (Figure 2A, rows). The comparison of COMBAT Stroke with treatment decision based on manually outlined mismatch statistics demonstrated excellent agreement; 93% sensitivity and 95% specificity, 97% positive predictive value and 87% negative predictive value (Figure 2B).

### Volumetric agreement

**Figure 3:**
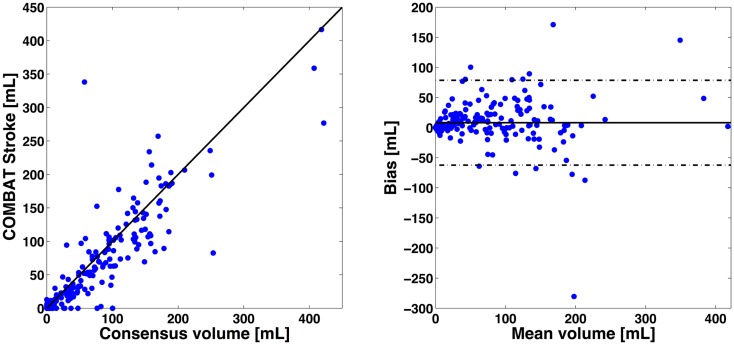
**Volumetric comparisons of mismatch delineations**. On left, *x*-axis represents the expert consensus mismatch volume and *y*-axis represent the mismatch volume estimates by the automatic approaches. On right, *x*-axis represents the average mismatch volume between expert consensus and automatic approaches. The *y*-axis represent the difference in mismatch volume between expert consensus and automatic approaches. The Spearman correlation was *R*^2^ = 0.89 [CI: (0.88, 0.91)] for COMBAT Stroke compared with the manual outlining. The mean difference in volume between manual and COMBAT Stroke outlining was 8 mL (SD: 35 mL). Solid line indicates the mean between the two compared methods and dashed lines indicate mean difference ±2 SD.

The correlation between PWI-DWI mismatch volumes using the automatic algorithm and manual outlines performed by experts was excellent, with Spearman *R* = 0.89 [CI: (0.86, 0.91)] (Figure 3). The mean difference in mismatch volume between the manual and automatic approach was 8 mL (SD: 35 mL), indicating an overall underestimation by COMBAT Stroke (Figure 3). We observed a significant difference in median mismatch volume in the manual outlines estimated by COMBAT Stroke between patients scanned on a 1.5T [median mismatch volume = 80 mL (27, 135), *p* < 0.01] versus 3T [median mismatch volume = 29 mL (1, 95)] MRI. Likewise, this difference was apparent in COMBAT Stroke estimations; 1.5T: median mismatch volume = 62 mL (13, 106), *p* < 0.01, 3T: median mismatch volume = 15 mL (0, 102).

### Acute decision parameters

**Figure 4:**
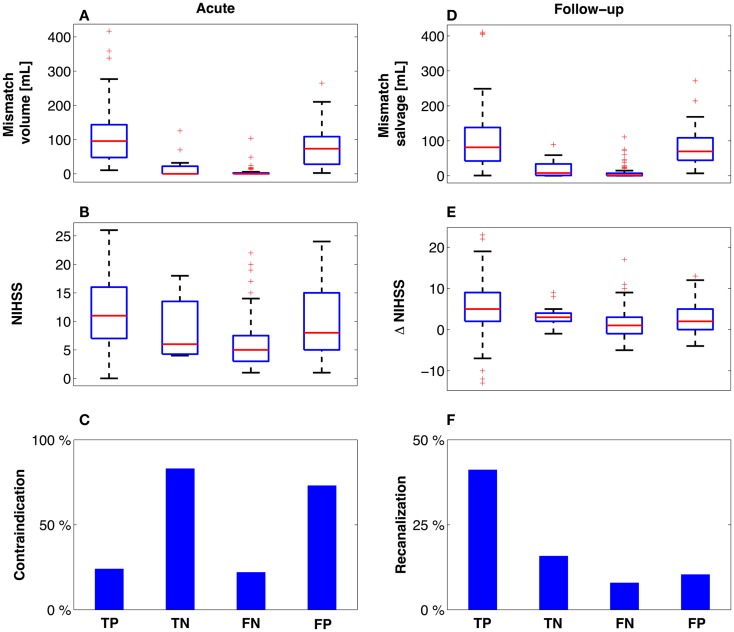
**Descriptive variables at presentation and follow-up**. Using the confusion matrix established, descriptive variables are presented related to the mismatch **(A,D)** and degree of neurological impairment **(B,E)**. Notably, patients who did not meet radiographic treatment criteria based on COMBAT Stroke **(C)** and did not receive treatment (TN) had equal outcomes **(E,F)** compared to the patients who similarly did not meet radiographic treatment criteria, yet received thrombolysis (FN). Red line = median, box = 25th–75th percentiles, bars image most extreme data points not considered outliers and + depict outliers plotted individually.

The patients in the TP group (112 patients actually receiving thrombolytic treatment where COMBAT stroke determined them to be potential candidates) had significantly higher median mismatch volume [96 mL (48, 144)], compared with TN [12 patients, median = 0 mL (0, 22), *p* < 0.001], FN [74 patients, median = 0 mL (0, 3), *p* < 0.001] and the FP [30 patients, median = 73.4 mL (28, 109), *p* = 0.04] (Figure 4A). Additionally, the NIHSS score was significantly higher in the TP group [median = 11 (7, 16)] compared with TN [median = 6 (4.25, 13.5), *p* < 0.001], FN [median = 5 (3, 7.5), *p* < 0.001] and FP [median = 8 (5, 15), *p* < 0.001] (Figure 4B). Contraindications to thrombolysis were present in 83% of the patients in the TN group and in 73% in the FP group. Among the patients in the TP and FN groups, contraindications were present in 24 and 23% of cases, respectively (Figure 4C). A summary of acute decision parameters statistics and significance levels are given in Table 2 for TN and FN group.

**Table 2:** **Summary of acute decision parameters and clinical outcome parameters for TN and FN groups**.

	TN	FN	*p*
Mismatch	0 mL (0, 22)	0 mL (0, 3)	0.26
Mismatch salvage	8.2 mL (1, 34)	1.5 mL (0, 7)	0.06
Acute NIHSS	6 (4.25, 13.5)	5 (3, 7.5)	0.4
ΔNIHSS	3 (2, 4)	1 (−1, 3)	0.8
Contraindications	83%	23%	N/A
Recanalization	15%	8%	N/A
3-Months mRS	2 (1, 3.75)	1 (0, 1.5)	<0.01
3-Month NIHSS	2 (0.25, 6.5)	0 (0, 1)	<0.01

### Clinical outcome

The 112 patients in the TP group demonstrated the largest mismatch salvage [median = 84.8 mL (42, 138)] and significantly greater salvage than in the TN [median = 8.2 mL (1, 34), *p* < 0.001] and FN groups [median = 1.5 mL (0, 7), *p* < 0.001]. No significant difference in the volume of salvaged tissue was observed between the FP group [median = 73.4 mL (44, 109), *p* = 0.05] and TP (Figure 4D). The neurological improvement (assessed by reduction in NIHSS score at 24 h) was significantly better for the TP group [median = 5 (2, 9)] compared with each of the other three groups [TN: median = 3 (2, 4), *p* = 0.03; FN: median = 1 (−1, 3), *p* < 0.001; FP: median = 2 (0, 5), *p* = 0.01] (Figure 4E). Routine 24 h MR angiography demonstrated recanalization in 42% of the patients in the TP group, 15% of the TN group, 8% of the FN group, and 10% of the patients in the FP group (Figure 4F). A summary of 3 month outcomes (mRS and NIHSS score) are given in Table 2 for TN and FN group.

### CART analysis

**Figure 5:**
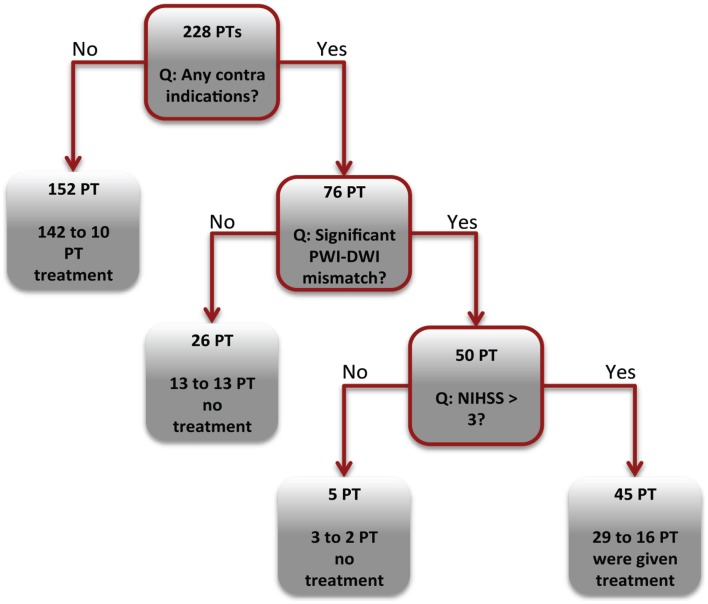
**Decision tree analysis**. The CART tree illustrates the classification of patients (PT) into thrombolysis or supportive treatment. The boxes with red borders indicate a binary question related to the acute clinical or imaging markers. The boxes without red boarders denote patient classification into a treatment group.

According to CART analysis, clinicians based their treatment decisions on a multitude of factors, but primarily on the presence/absence of a contraindication (Figure 5). The majority of the patients who had no contraindications (152 of 228) were treated with thrombolysis (*n* = 142). If the patient had one or more contraindications, the clinician involved the PWI-DWI mismatch ratio and mismatch volume as a secondary parameter in the clinical decision-making. Finally, neurological assessment quantified by NIHSS score appeared to be the least influential parameter in the regression tree.

## Discussion

This study reports a 93% sensitivity and 95% specificity between automated patient triaging by COMBAT Stroke and treatment decisions rendered by manually outlined mismatch statistics. Patients with significant mismatch volume and ratio (TP and FP groups) at the time of acute MRI imaging demonstrated higher mismatch salvage compared with those who hadn’t significant mismatch, regardless the clinical treatment decision (Figure 4).

Not surprisingly, there was a poor agreement between COMBAT Stroke and the actual clinical decision made (Figure 2A). This was most notable in patients that did not receive thrombolysis. In that group, there were only 12 cases where there was agreement between clinical and automated decision-making (29% specificity). In contrast, in 74 cases the clinicians decided to administer thrombolysis when COMBAT Stroke decided against (60% sensitivity). This poor correlation reflects the many different variables associated with clinical care and treatment decisions.

Importantly, our results indicate that the patients who did not meet mismatch treatment criteria based on COMBAT Stroke and did not receive treatment (TN) had higher rates of recanalization (15%), larger reduction in NIHSS score (median = 3), and greater mismatch salvage (median = 8.2 mL) compared to the patients who similarly did not meet mismatch treatment criteria, yet received thrombolysis (FN). The analysis of 3 months mRS and NIHSS score outcomes revealed that patients in FN group had significantly better functional recovery compared to TN group (Table 2). It should be emphasized that the patients in FN group had, in general, the mildest grade of neurological deficit at admission, Figure 4B. Therefore, selection bias may be a confounding factor in the good functional recovery observed in this group. Overall, this data supports a need for greater emphasis on the contribution of strict radiographic criteria in the decision-making process. Use of an automated software system such as COMBAT Stroke may provide rapid and accurate information to objectively contribute to the clinical decision.

In general, the patients COMBAT Stroke identified for potential treatment and were treated had significantly better neurological improvement, mismatch salvage, and higher rates of recanalization compared to the other groups. This indicates the importance of the size of the mismatch and the ratio of the potentially salvageable and infarcted tissue with outcome following thrombolysis (18). Our data suggests that the volumetric assessment of the mismatch provided by COMBAT Stroke, in conjunction with the clinical assessment, is potentially a decision-support tool for rapid and standardized stroke management.

To further investigate the factors that influence decision to perform thrombolysis, a CART analysis was performed. About 93% of patients without contraindications were administered thrombolysis (Figure 5). Among the treated patients, 15 and 12% of TP and FN groups had contraindication to treatment, in contrast to the 83 and 78% in the TN and FP groups. It could be suggested that the clinicians in our cohort followed an “aggressive” treatment strategy by administering thrombolysis in the presence of generally accepted contraindications (19, 20). This is not entirely unexpected, as each patient’s care must be individualized, and a risk-benefit ratio of treatment cannot be accurately predicted or captured from our database variables alone (21).

The main limitations of this study are those inherent in any retrospective design. We attempted to overcome selection bias by utilizing a consecutive cohort of ischemic stroke patients from two detailed multi-center databases. Variations in clinical practices between, and even within, different hospitals are not possible to account for and can influence the rates of intervention and clinical outcome (22, 23). Furthermore, individual aspects of patient care such as the patient’s own preexisting wishes regarding treatment are not typically captured in a retrospective analysis. Unfortunately, the balance between actively and supportively treated patients was highly skewed, thus drawing conclusions by comparing the different groups should be done cautiously. The main technical limitations in this study are the numerous automatic post-processing steps in COMBAT Stroke algorithm. For instance, the co-registration is fully automated and in cases where the co-registration is not optimal, the determination of mismatch volume and mismatch salvage is incorrect. To avoid incorrect estimation of the radiographic parameters, we manually checked all automated steps in the COMBAT Stroke algorithm.

Our study identifies many aspects of investigating an automated clinical decision-support tool that can be further investigated in a prospective fashion. These include any causal effect on neurological outcome, radiographic outcome, and even “door-to-needle” time. The automated process of mismatch volumetric calculation may also prove valuable in the growing population of patients that are treated beyond the typical treatment time window based on dynamic imaging characteristics (24–28).

## Conclusion

COMBAT Stroke may help to not only identify patients that are potential candidates for thrombolysis, but also to exclude patients that are unlikely to experience benefit. COMBAT Stroke, in addition to clinical assessment, may provide a decision-support tool for a fast, efficient, and standardized clinical decision-making in acute ischemic stroke.

## Conflict of Interest Statement

The authors declare that the research was conducted in the absence of any commercial or financial relationships that could be construed as a potential conflict of interest.
